# Work–Family Conflict, Emotional Responses, Workplace Deviance, and Well-Being among Construction Professionals: A Sequential Mediation Model

**DOI:** 10.3390/ijerph17186883

**Published:** 2020-09-21

**Authors:** Yan Chen, Feilian Zhang, Yan Wang, Junwei Zheng

**Affiliations:** 1 School of Civil Engineering, Central South University, Changsha 410075, China; 2256@hutb.edu.cn (Y.C.); zfl@csu.edu.cn (F.Z.); 2 College of Computer and Information Engineering, Hunan University of Technology and Business, Changsha 410205, China; 3 Collaborative Innovation Center for Integration of Marine & Terrestrial Economics and Construction of Marine Silk Road, Guangxi University of Finance and Economics, Nanning 530007, China; wangyan@ujs.edu.cn; 4 Department of Construction Management, Kunming University of Science and Technology, Kunming 650500, China

**Keywords:** work–family conflict, negative affect, emotional exhaustion, deviant behavior, workplace well-being, construction professionals, affective events theory

## Abstract

Given the dynamic, complex, and highly demanding project environment, construction professionals are particularly likely to experience a high level of work–family conflict. Taking an emotional resource perspective and on the basis of affective events theory, this study tested negative affect and emotional exhaustion as sequential mediators between two directions of work–family conflict and workplace well-being or deviance behavior. The theoretical model was examined using data collected at two time points from 143 construction professionals through regression analysis and bootstrapping. The results indicate that work–family conflict was positively related to deviant behavior and negatively related to workplace well-being. The findings demonstrate that the mediation effects of emotional exhaustion between work–family conflict and workplace well-being or deviant behavior were significant and that the sequential mediating effects of negative affect and emotional exhaustion in the relationship between work–family conflict and workplace well-being or deviant behavior were significant. Moreover, different impacts of work interference with family and family interference with work on job-related attitudes and behavior were observed. These findings highlight the importance of emotional experience to understand the negative impact of work–family conflict in the temporary project context.

## 1. Introduction

Construction project work is characterized by complexity, uncertainty, and ambiguity [1,2]. Project managers and construction professionals experience long working hours and must complete complex tasks to meet the requirements of project goals [3,4], which result in high levels of work–family conflict (WFC) [5]. WFC refers to a form of inter-role conflict [6], that occurs when the demands of functioning in the work and family domains are incompatible [7]. This conflict can manifest itself in either of two directions: work can interfere with family life (work-to-family conflict, WIF) and family life can interfere with the workplace (family-to-work conflict, FIW) [8,9].

Prior work on project management or construction management has focused on work–family conflict issues and has examined the consequences of conflict, including burnout [10,11], turnover intention [12], sleep problems [13], organizational commitment [5,14], and project citizenship behavior [5]. A great deal of research on permanent organizations has focused on the detrimental effects of work–family conflict on mental health (e.g., strain [15], subjective well-being [8]), work attitudes (e.g., job satisfaction and life satisfaction [16,17]), and behaviors (e.g., aggressive behavior [18]). The intensive and chronic work–family conflict suffered by construction professionals, such as construction site managers, on-site estimators or operatives, and civil or structural engineers [19,20], is thus likely to undermine their mental health and increase their engagement in problem behaviors. Construction professionals have been considered one of the most stressed-out groups in the construction industry [21]. Nevertheless, few studies have undertaken an examination of the mechanism underlying the effect of work–family conflict on mental health and problem behavior in the context of temporary construction projects. 

Furthermore, studies linking work–family conflict with relevant outcomes mainly draw on conservation of resources (COR) theory [22], role theory [6], and the job demands–resource (JD-R) model [23]. These theoretical perspectives and models assume that work–family conflict is the stressor [8,24] that depletes resources and generates strain, leading to negative outcomes [22,25]. The accumulated strain can decrease well-being [8], and resource depletion can increase workplace deviance [26]. Specifically, well-being refers to one’s subjective experience of happiness [27], and workplace deviance has been believed to be voluntary and pervasive behavior that violates the norms or expectations in the organizational context [28]. However, previous research has always focused on the direct effects of work–family conflict on these outcomes [18,29], while scant attention has been paid to empirical examination of the mechanism that link work–family conflict, workplace deviance, and well-being. Thus, there is a need to open this “black box” to address the interconnections among work–family conflict, workplace deviance, and well-being for construction professionals in order to ensure the project goal achievement and facilitate project success. A major purpose of this research is thus to extend conservation of resource theory to uncover the mechanisms by which work–family conflict influences deviant workplace behavior and well-being within the context of construction projects.

Affective events theory (AET) focuses on workplace events as the causes of affective experience and demonstrates that affective responses may influence judgement or satisfaction toward one’s job to form workplace behaviors [30]. Research on AET to date has highlighted the role of emotional experiences, between workplace events and problem behaviors (e.g., counterproductive behaviors [31]), as well as between affect and well-being [32]. On the basis of the tenets of AET, work–family conflict will result in increased problem behaviors and reduced well-being through a process mediated by the individual’s emotional experience [33]. The application of AET in this study begins with an examination of the impact of work–family conflict on accumulated negative affect, and then on emotional exhaustion (psychological state), and finally on workplace well-being (attitude) and deviance (behavior). The rationale is that work–family conflict is likely to give rise to negative affect [34], which depletes the individual’s limited emotional resources and thus engenders emotional exhaustion [18]. This in turn makes it difficult for individuals to control their attitudes and behaviors in order to conform to workplace demands and norms, even to the point of leading them to engage in deviant behavior or attenuating their well-being in the workplace. Another purpose of this research is thus to invoke an emotional resource perspective to demonstrate negative affect and emotional exhaustion as the key emotional experiences through which the bidirectional work–family conflict influences the work attitude and behavioral outcome.

**Figure 1:**
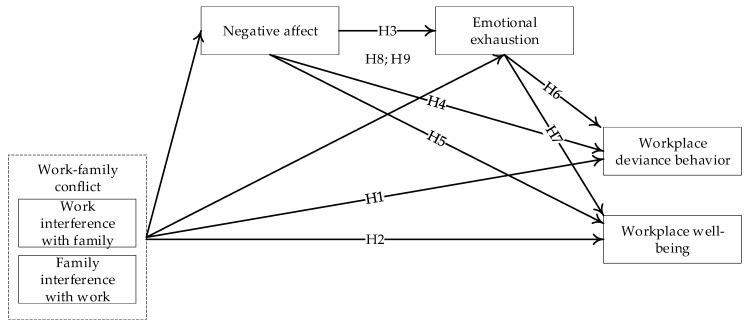
The conceptual model.

Figure 1 illustrates the conceptual model developed in this paper. This study makes three primary contributions to the literature. First, this study answers the call for more research on the affective implications of role conflict in the workplace [34,35]. It combines an emotional resource perspective with AET to establish a theoretical framework for understanding work–family conflict and its relationship to emotional experience. Second, this study introduces new theoretical explanations regarding the spillover effects of work–family conflict by testing workplace well-being and deviant behavior in the work and family domains as the results of that conflict. Issues about work–family conflict and positive behavioral outcomes (e.g., citizenship behavior [5]) have been explored in the project context. However, less research has investigated the potential impact of work–family conflict on problem behaviors such as deviant behavior in the project context. Third, this research extends work–family research on the impacts of bidirectional work–family conflict into the context of construction projects. Within the Eastern context (i.e., China), people may prioritize work over family [5,36], and thus WIF is likely to cause less stressed problems compared to FIW [37,38]. Therefore, the difference in the impacts of two directions of work–family conflict can also be examined in this research to demonstrate which one (i.e., WIF or FIW) would exert greater negative influences on work attitude and behavior.

## 2. Theory and Hypotheses 

### 2.1. Work–Family Conflict, Deviant Behavior, and Workplace Well-Being

Workplace deviant behavior (WDB) is defined as voluntary behavior that violates important norms in the organization and then threatens the well-being of colleagues or the organization [39]. Workplace deviance includes several forms. It can be directed at the organization (e.g., production deviance), including leaving early or taking immoderate breaks, or it can be interpersonal deviance directed at organizational members (e.g., interpersonal aggression), including losing one’s temper at work or acting rudely [28,39]. 

Considering the dynamic and complex work environment of construction sites, construction professionals are likely to experience long working hours [3,4], after which they may have insufficient energy and time to balance their responsibilities in the work and family domains [5]. On the basis of to the results of a meta-analysis, Allen et al. [17] demonstrated that bidirectional work–family conflict (i.e., WIF and FIW) are associated with both work-related and family-related stress. According to the tenets of COR theory, stress creates resource loss and gives rise to the depletion of energy and effort [22]. However, Krischer et al. [40] stated that withdrawal behavior and deviant behavior are ways in which employees conserve their resources and reduce resource loss in response to workplace stress. The experience of WFC depletes the individual’s resources and results in deviant behavior in the workplace as an approach to maintain or conserve resources [41]. Thus, construction professionals who spend energy and effort on the fulfillment of work and family roles are more likely to slack off at work to protect their resources (e.g., working slowly) or replenish their resources (e.g., working on a personal matter, or taking a longer break). Work–family conflict can thus be taken as a precursor to voluntary deviant behavior [42]. On this basis, the following hypotheses are proposed:

**Hypothesis** **1.** *Work–family conflict is positively related to deviant behavior in construction projects.*

**Hypothesis** **1a.** *Work interference with family is positively related to deviant behavior in construction projects*.

**Hypothesis** **1b.** *Family interference with work is positively related to deviant behavior in construction projects.*

Workplace well-being (WWB) comprises job satisfaction and work-related experience [43]. It is different from subjective well-being, which focuses on the life satisfaction or the overall evaluation of life quality [44]. In their meta-analyses, Allen et al. [17] and Amstad et al. [16] demonstrated a negative correlation between WFC and job satisfaction. When individuals perceive a high level of WIF or FIW, their energy and effort are depleted, and they may then become resentful or attribute their troubles to the job or organization [45]. In addition, WFC is a stressor that can lead to discontent with work and decrease job satisfaction or well-being in the workplace [46]. The negative association between WFC and job satisfaction or workplace well-being has been evidenced for seafarers [46], correctional officers [47], and hotel employees [48]. Owing to the stressful environment of construction projects, this study also proposes that construction professionals experience WIF or FIW, which results in negative workplace well-being. Thus, the following hypotheses are proposed:

**Hypothesis** **2.** *Work–family conflict is negatively related to workplace well-being in construction projects.*

**Hypothesis** **2a.** *Work interference with family is negatively related to workplace well-being in construction projects.*

**Hypothesis** **2b.** *Family interference with work is negatively related to workplace well-being in construction projects.*

### 2.2. Negative Affect and Emotional Exhaustion

Affect is defined as an individual’s experience of their moods and emotions [33]. Emotional exhaustion is one dimension of job burnout and is defined as a psychological state of emotional and physical depletion created by work-related reasons [49]. Thus, negative affect and emotional exhaustion are different concepts or constructs that have distinct impacts on the evaluation of life quality and work [50]. According to COR theory [51,52], negative affect represents a loss and lack of resources. Negative affect results in resource depletion and is positively associated with emotional exhaustion [53]. The individual that experiences a high level of negative dispositional affectivity is more likely to be distressed, and emotional exhaustion is related to negative feelings of being exhausted by his/her work [54]. Thus, the following hypothesis is proposed:

**Hypothesis** **3.** *Negative affect is positively related to emotional exhaustion.*

### 2.3. The Mediating Role of Negative Affect

WFC can be not only assessed as the perception of the average interference or roles conflict level, but also operationalized as events [55]. According to AET [30], specific events in the work environment generate discrete emotional responses, which then result in work attitudes and behaviors. High levels of WIF and FIW give rise to individual negative affective states [34,48]. The negative experience then has a negative impact on work behavior and well-being in the workplace [56]. 

Deviant behavior is a behavioral response to strain in a stressful work environment [57]. According to the stressor–emotional model [58], workplace events or conditions may not directly result in the outcome behaviors, but first give rise to negative feelings. Thus, given the increasing uncertainty and complexity of the changing environment of construction projects, work–family conflict can be taken as the stressor that would trigger negative affect in the form of anger or frustration [59]. The negative affect experienced by construction professionals can then lead to detrimental effects on themselves and the organization, such as the harmful deviant behavior. Negative affect plays a pivotal mediating role in the process by which stressors (e.g., WIF and FIW) give rise to behavioral reactions (e.g., workplace deviant behavior). The following hypotheses are thus proposed:

**Hypothesis** **4.** *Work–family conflict has a significant indirect effect on workplace deviant behavior via negative affect.*

**Hypothesis** **4a.** *Work interference with family has a significant indirect effect on workplace deviant behavior via negative affect.*

**Hypothesis** **4b.** *Family interference with work has a significant indirect effect on workplace deviant behavior via negative affect.*

According to AET, workplace well-being or job satisfaction is an attitude engendered by an individual’s affect [33]. AET suggests that accumulated affective experiences in the workplace shape one’s job-related attitudes [30]. Kafetsios and Zampetakis [60] have provided evidence that negative affect has a negative impact on job-related attitudes such as job satisfaction. According to the meta-analytic results, the absence of negative affect was positively associated with well-being [61]. Thus, WIF or FIW may cause an affective response or experience in a stressful project environment, and the negative affectivity would then reduce job-related attitudes such as workplace well-being toward the intensive work. The following hypotheses are thus proposed:

**Hypothesis** **5.** *Work–family conflict has a significant indirect effect on workplace well-being via negative affect.*

**Hypothesis** **5a.** *Work interference with family has a significant indirect effect on workplace well-being via negative affect.*

**Hypothesis** **5b.** Family interference with work has a significant indirect effect on workplace well-being via negative affect.

### 2.4. The Mediating Role of Emotional Exhaustion

According to the extant literature on occupational health, WIF and FIW are positively related to job burnout [16,17]. Emotional exhaustion is a marker of resource depletion, which is caused by work demands or stressors [55,62]. When emotionally exhausted by work events, resource-depleted employees are less likely to conserve their self-regulatory resources to maintain their behaviors to achieve high performance [18,63]. Emotionally exhausted employees might engage in workplace deviant behavior as their response to resource depletion to protect their limited resources [64]. COR theory suggests that the passive behavior is an approach to conserve the individual’s remaining resources in a stressful environment [40]. According to AET, workplace deviant behavior represents the affect-driven behavior to response to the affect in the context of the workplace [30,65]. Thus, emotional exhaustion may play a mediating role between events or stressors (e.g., WFC) and affect-driven behavior or resource protecting behaviors (e.g., deviant behavior). The hypotheses proposed are as follows:

**Hypothesis** **6.** *Work–family conflict has a significant indirect effect on workplace deviant behavior via emotional exhaustion.*

**Hypothesis** **6a.** *Work interference with family has a significant indirect effect on workplace deviant behavior via emotional exhaustion.*

**Hypothesis** **6b.** *Family interference with work has a significant indirect effect on workplace deviant behavior via emotional exhaustion.*

Job resources and personal resources are predictors of employee well-being [66]. Emotional exhaustion represents the resource depletion that results from emotional experiences at work [18,62], and the drain of resources can decrease the cost of work engagement and well-being in the workplace. Skaalvik et al. [67] as well as Schaufeli and Salanova [68] also stated that emotional exhaustion is negatively related to job satisfaction and positively related to turnover intention. Workplace well-being or job satisfaction can also be considered to be an individual’s attitude toward their job, while emotional exhaustion is an indication of their emotional experience in the workplace. From the perspective of AET [30], work events (e.g., WIF or FIW) give rise to emotional experiences (e.g., emotional exhaustion), which in turn affect job-related attitudes (e.g., workplace well-being). Thus, the following hypotheses are proposed:

**Hypothesis** **7.** *Work–family conflict has a significant indirect effect on workplace well-being via emotional exhaustion.*

**Hypothesis** **7a.** *Work interference with family has a significant indirect effect on workplace well-being via emotional exhaustion.*

**Hypothesis** **7b.** *Family interference with work has a significant indirect effect on workplace well-being via emotional exhaustion.*

### 2.5. The Mediating Roles of Negative Affect and Emotional Exhaustion

Overall, the above-mentioned hypotheses suggest that construction professionals’ WIF or FIW are likely to increase their deviant behavior and reduce their well-being in the construction projects by giving rise to negative affect, which then causes emotional exhaustion. This sequence is in line with the resource depletion mechanism posited by COR theory, and also corresponds with the formation of affect-driven behavior and job-related attitudes caused by work events and emotional responses based on AET. Thus, this study suggests that the effect of WFC on workplace deviant behavior or well-being will be mediated by negative affect and then emotional exhaustion. The following hypotheses are proposed:

**Hypothesis** **8.** *Work–family conflict has a significant indirect effect on workplace deviant behavior via negative affect and then emotional exhaustion.*

**Hypothesis** **8a.** *Work interference with family has a significant indirect effect on workplace deviant behavior via negative affect and then emotional exhaustion.*

**Hypothesis** **8b.** *Family interference with work has a significant indirect effect on workplace deviant behavior via negative affect and then emotional exhaustion.*

**Hypothesis** **9.** *Work–family conflict has a significant indirect effect on workplace well-being via negative affect and then emotional exhaustion.*

**Hypothesis** **9a.** *Work interference with family has a significant indirect effect on workplace well-being via negative affect and then emotional exhaustion.*

**Hypothesis** **9b.** *Family interference with work has a significant indirect effect on workplace well-being via negative affect and then emotional exhaustion.*

## 3. Methods

### 3.1. Sample and Procedure

Data were collected via the online questionnaire survey in this study. The construction professionals working on construction projects or construction enterprises located in China were initially invited to join in our study. The website link was sent to construction professionals or companies holding construction projects that consented to participate in this study through the email or WeChat. The announcement included the beginning of the survey was informed that all answers would be kept confidential and used only for research purposes. The respondents were recruited to voluntarily participate in the survey at two time points within approximately 2 months. This time interval was long enough to separate the assessments of the independent variables and mediators from the dependent variables [69,70]. 

At Time 1, we invited 500 construction professionals to report the demographic information such as gender, age, educational level, and organizational tenure. They were also required to rate their level of work–family conflict and their affect in the workplace. Among them, 348 professionals responded to our survey (69.60%). At Time 2, the respondents who responded at Time 1 were asked to report their evaluation of their emotional exhaustion and workplace deviant behavior they may have performed, as well as their perception of well-being in the workplace. We received responses from 177 construction professionals (50.86%). After matching the responses from two time points and deleting the invalid sample with missing data, the final valid sample size comprised 143 respondents.

We compared our final sample (i.e., 143 professionals) with an initial sample (i.e., 348 professionals) in terms of the gender, age, education, work–family conflict, and negative affect, which are the variables shared by both samples. Analyses of variance examination demonstrated that there were no significant differences between the two groups on age (*F* = 1.720, *n.s.* (nonsignificant)*)*, education (*F* = 2.878, *n.s.*), tenure (*F* = 0.587, *n.s.*), work–family conflict (*F* = 1.943, *n.s.*), and negative affect (*F* = 1.095, *n.s.*).

As indicated in Table 1, the final valid respondents covered various construction professionals, including construction site managers (14.7%), on-site operatives (33.6%), civil or structural engineers (23.8%), cost engineers (20.9%), and others (7.0%). Most of these construction professionals worked for construction companies (79.0%), while others were from real estate companies (9.8%), design institutes (1.4%), consulting companies (5.6%), and others (4.2%). Of the valid sample, 30 were female (21.0%) and 113 were male (79.0%). The average age was 30.77 years (SD = 7.35), and the average length of organizational tenure was 8.03 years (SD = 7.11). With regard to educational level, 8 had a high school education or below (5.6%), 129 were junior college graduates or had undergraduate degrees (90.2%), and 6 had postgraduate degrees or above (4.2%). 

### 3.2. Measures 

Existing and verified measures used in the prior literature were applied in this survey. Correct translation was confirmed by translation and back-translation between English and Chinese [71,72]. All measure items were assessed using a five-point Likert scale ranging from “1 = strongly disagree” to “5 = strongly agree”.

*Work*–*family conflict* (WFC) was measured at Time 1 using a two-dimensional scale developed by Grandey et al. [55] and adapted from Zhao et al. [73] measuring work interference with family (WIF; six items such as “I spend so much time working that I am unable to get much done at home”) and family interference with work (FIW; five items such as “The demands of my family life limit the number of hours I’m able to work”). The total Cronbach’s *α* was 0.912. The Cronbach’s *α* coefficients of WIF and FIW were 0.953 and 0.951, respectively.

*Negative affect* (NA) was assessed at Time 1 using a five-item scale developed by Watson et al. [74] and adapted by Wong et al. [75]. The construction employees were required to evaluate their affect in the past month using five items, namely, “unhappy”, “jittery”, “troubled”, “scared”, and “irritable”. The Cronbach’s *α* was 0.921.

*Emotional exhaustion* (EE) was evaluated at Time 2 using a five-item scale developed by Maslach [49] and revised for the Chinese context by Li and Shi [76]. A sample item is “I feel emotionally drained by my work”. The Cronbach’s *α* was 0.935.

*Workplace deviant behavior* (WDB) was assessed at Time 2 using a two-dimensional scale developed by Stewart et al. [77], including production deviance (PD; five items such as “Put little effort into work”) and personal aggression (PA; five items such as “Lost my temper while at work”). The Cronbach’s *α* coefficients of PD and PA were 0.896 and 0.881, respectively. The total Cronbach’s *α* was 0.909.

*Workplace well-being* (WWB) was evaluated at Time 2 using a six-item scale developed by Zheng et al. [43]. A sample item is “I find real enjoyment in my work”. The Cronbach’s *α* was 0.936.

*Control variables*. The demographic characteristics, including gender (0 = female, 1 = male), educational level (1 = high school or below, 2 = junior college or undergraduate, 3 = postgraduate or above), and age (years) were control variables in this study.

Moreover, to evaluate the causal relationships between the key studied variables and to eliminate alternative explanations, we also included emotional exhaustion (Cronbach’s *α* = 0.933), workplace deviance behavior (Cronbach’s *α* = 0.909), and workplace well-being (Cronbach’s *α* = 0.933) collected at Time 1 as control variables. The attentiveness from the positive affect measurement developed by Watson and Clark [74] and adapted from Rodell and Judge [78] collected at Time 1 was also controlled for. The specific items of “*attentive*, *alert*, and *determined*” for *Attentiveness* were assessed, and the Cronbach’s *α* was 0.761.

## 4. Results

### 4.1. Preliminary Analysis

Confirmatory factor analyses (CFAs) were conducted using the chi-squared (*χ*^2^) test through MPLUS (version 7.0) [79] to examine the proposed model. Multiple goodness-of-fit indices, including the ration of chi-squared and degree of freedom (*χ*^2^*/df*), root-mean-square error of approximation (RMSEA), standardized root-mean-square residual (SRMR), comparative fit index (CFA), and Tucker–Lewis index (TLI), were applied to assess different aspects of model fit in this study. *χ*^2^*/df* and SRMR provide the examples of absolute fit; RMSEA provides an example of parsimony fit; CFI and TLI demonstrate the comparative fit [80].

As indicated in Table 2, the expected model with six factors demonstrated a better, more acceptable fit to the valid sample data (*χ*^2^*/df* = 1.681, CFI = 0.916, TLI = 0.908, RMSEA = 0.069, SRMR = 0.060). A criterion of 0.90 has been recommended as fit for CFI and TLI [81], and 0.08 has been recommended as the acceptable fit for RMSEA and SRMR [81,82]. Compared to the hypothesized six-factor model, the CFA results of alternative models showed increased values of *χ*^2^*/df*, RMSEA, and SRMR and decreased values of CFI and TLI. The CFA results also demonstrated good discriminant validity for the studied variables.

Table 3 illustrates the descriptive statistics and inter-correlation coefficients among the study variables. As indicated in Table 3, the intercorrelations were in agreement with the hypotheses. Work interference with family and family interference with work showed positive correlations with negative affect (*r* = 0.470, *p* < 0.001; *r* = 0.338, *p* < 0.001). Negative affect showed a positive correlation with emotional exhaustion (*r* = 0.432, *p* < 0.001).

### 4.2. Hypothesis Testing

Table 4 illustrates the analysis of direct effects. Hypothesis 1 concerns the direct effects of work–family conflict on workplace deviant behavior. As shown by the results of Model 3 and Model 3ab in Table 4, work–family conflict is positively related to workplace deviant behavior (*β* = 0.222, *p* < 0.05) when controlling the workplace deviant behavior at Time 1. This result yields support for Hypothesis 1. WIF is positively but insignificantly related to workplace deviant behavior (*β* = 0.079, *p* > 0.05), while FIW is positively and significantly related to workplace deviant behavior (*β* = 0.222, *p* < 0.05). Thus, Hypothesis 1a was not supported, but Hypothesis 1b was supported.

Hypothesis 2 predicted a negative relationship between work–family conflict and workplace well-being. As indicated in Model 4 and Model 4ab in Table 4, work–family conflict is negatively related to workplace well-being (*β* = −0.142, *p* < 0.05) when controlling the workplace well-being at Time 1, supporting Hypothesis 2. Further, WIF was also found to be negatively related to workplace well-being (*β* = −0.213, *p* < 0.01), supporting Hypothesis 2a. However, FIW was found to be insignificantly related to workplace well-being (*β* = 0.073, *p* > 0.05), and thus Hypothesis 2b was unsupported.

Hypothesis 3 suggested a positive relationship between negative affect and emotional exhaustion. As indicated by Model 2 and Model 2ab in Table 4, negative affect at Time 1 is positively related to emotional exhaustion at Time 2 (*β* = 0.194, *p* < 0.05, Model 2; *β* = 0.201, *p* < 0.05, Model 2ab) when controlling for work–family conflict or two dimensions of work–family conflict and the emotional exhaustion at Time 1, supporting Hypothesis 3.

Additionally, as indicated by Model 1 and Model 1ab in Table 4, when controlling attentiveness that reflects the positive affect, work–family conflict is positively related to negative affect (*β* = 0.478, *p* < 0.001). WIF is positively and significantly related to negative affect (*β* = 0.379, *p* < 0.001), and FIW is also positively and significantly related to negative affect (*β* = 0.207, *p* < 0.001).

The bootstrap method and confidence interval (CI) were used to conduct mediation analysis through MPLUS [79,83]. With regard to the criterion of confidence interval, if zero is not between the lower and upper level of 95% confidence interval, then the null hypothesis can be rejected and we can claim that the indirect effect is significant at 95% level [84]. The results were presented in Table 5. As shown by the results of Model 5, the indirect effect of work–family conflict on workplace deviant behavior via negative affect was insignificant (0.029, *p* > 0.05, 95% CI −0.058; 0.102]), and thus Hypothesis 4 was unsupported. The indirect effect of work–family conflict on workplace well-being via negative affect was also insignificant (−0.033, *p* > 0.05, 95% CI −0.177; 0.092]), and thus Hypothesis 5 was unsupported. Furthermore, as shown by the results of Model 6 in Table 5, the indirect effects of WIF and FIW on workplace deviant behavior via negative affect were insignificant (0.022, *p* > 0.05, 95% CI [−0.030; 0.064]; 0.014, *p* > 0.05, 95% CI = [−0.013; 0.057], containing 0), and thus Hypotheses 4a and 4b were unsupported. The indirect effects of WIF and FIW on workplace well-being via negative affect were also insignificant (−0.016, *p* > 0.05, 95% CI [−0.011; 0.052]; −0.010, *p* > 0.05, 95% CI [−0.068; 0.040]) and thus Hypotheses 5a and 5b were unsupported.

As indicated in Model 5 of Table 5, the indirect effect of work–family conflict on workplace deviant behavior via emotional exhaustion was significant (0.127, *p* < 0.01, 95% CI [0.054; 0.247]); therefore, Hypothesis 6 was supported. The indirect effect of work–family conflict on workplace well-being via emotional exhaustion was also significant (−0.091, *p* < 0.05, 95% CI [−0.203; -0.017]), and thus Hypothesis 7 was supported. According to the results of Model 6 in Table 5, the indirect effect of WIF on workplace deviant behavior via emotional exhaustion was significant (0.094, *p* < 0.01, 95% CI [0.043; 0.182]), but the indirect effect of FIW on workplace deviant behavior via emotional exhaustion was not significant (0.032, *p* > 0.05, 95% CI [−0.030; 0.107]), and thus Hypothesis 6a was supported while Hypothesis 6b was unsupported. The indirect effect of WIF on workplace well-being via emotional exhaustion was also significant (−0.061, *p* < 0.05, 95% CI [−0.146; −0.005]), however, the indirect effect of FIW on workplace well-being via emotional exhaustion was insignificant (−0.020, *p* > 0.05, 95% CI [−0.085; 0.015]). Therefore, Hypothesis 7a was supported and Hypothesis 7b was unsupported.

Hypotheses 8 and 9 suggest that there are chain mediation effects of negative affect and emotional exhaustion. According to Model 5 in Table 5, the indirect effect of work–family conflict on workplace deviant behavior via negative affect and emotional exhaustion was significant (0.049, *p* < 0.05, 95% CI = [0.019; 0.109]). The indirect effect of work–family conflict on workplace well-being via negative affect and emotional exhaustion was also significant (−0.035, *p* < 0.05, 95% CI [−0.089; −0.009]). These results provide support for Hypotheses 8 and 9.

Additionally, the estimate of the indirect effect of WIF on workplace deviant behavior via negative affect and emotional exhaustion was significant (0.030, *p* < 0.05, 95% CI [0.011; 0.071], Model 6), and the indirect effect of FIW on workplace deviant behavior was also significant (0.019, *p* < 0.05, 95% CI [0.006; 0.052], Model 6). These findings suggest that negative affect and emotional exhaustion mediate the effect of work interference with family or family interference with work on workplace deviant behavior. Hypotheses 8a and 8b were therefore supported. Moreover, estimations of the indirect effects of WIF or FIW on workplace well-being via negative affect and emotional exhaustion were significant (−0.019, *p* < 0.05, 95% CI [−0.053; −0.004], Model 6; −0.012, *p* < 0.05, 95% CI [−0.042; −0.002], Model 6), providing support for Hypotheses 9a and 9b.

In general, the results of hypothesis testing are summarized in Table 6.

## 5. Discussion

The aim of this study was to examine the processes by which WIF and FIW are related to workplace well-being and deviant behavior in the context of construction projects using affective events theory and conservation of resources theory as the guiding theoretical models. The findings indicate that construction professionals with higher WIF or FIW are more likely to experience negative affect, which in turn increases their emotional exhaustion and finally increases their deviant behavior and reduces their well-being in the workplace. These findings provide meaningful implications for theory and practice.

### 5.1. Theoretical Implications

This study has several theoretical implications. First, it adds to the body of work–family conflict research that examines the role of emotional responses and experience by testing the effects of negative affect and emotional exhaustion on WFC and job-related behaviors and attitudes in the workplace. Considering the importance of affective experience in the formation of attitudes and behaviors, Weiss [35] and Judge et al. [34] called for more refined research on the affective links between WFC and its consequences. Previous studies have taken affect to be an outcome of stress in the stressor–strain model [16,56], and the current study highlights the importance of affect in the relationships among WFC and job-related attitudes and behavior. However, the mediating effect of negative affect was insignificant, while the mediating effect of emotional exhaustion was significant. The findings indicate that the relationship between role conflict and well-being or passive behavior may be more complex than previously realized [56], and also reflect on the debate regarding whether negative affect or perceived stress are responsible for more of the variance between WFC and mental health. The results show that reduced workplace well-being and increased workplace deviant behavior may not be results of negative affect but instead follow from emotional exhaustion due to resource depletion and the demands of resource conservation [64]. This study complements the stressor–strain model in describing the relationship between WFC and strain by emphasizing the role of emotional experiences (i.e., the mediating effect of emotional exhaustion.)

Second, this study examined, from the perspectives of resources and affect, a sequential mediation model of the complex process by which WFC influences workplace deviant behavior and well-being. This is consistent with COR theory, which suggests that WFC can drain an individual’s resources in the work and family domains and thereby make construction professionals more likely to engage in problem behaviors, such as deviant behavior, and worsen job-related attitudes such as workplace well-being. This process is consistent with both AET and COR theory, which suggest that an individual’s attitudes or behaviors are caused by events or affective experience. These results are also consistent with Eby et al. [85] and Zhou et al. [56], who demonstrated that affective experiences appear to be the explanatory process underlying work–family conflict.

Third, this study enriches existing WFC research by extending it to the context of construction projects to assess and compare the impacts of WIF and FIW following the recommendations of Netemeyer et al. [86]. The existing literature concerning WFC in the context of construction projects has focused on its impact on burnout [10,11], turnover intention [12], psychological distress [13], and citizenship behavior [5]. However, less research has empirically investigated the asymmetrical effects of WIF and FIW in the context of construction projects. Previous studies have found that FIW potentially has a stronger effect on job-related outcomes than WIF [18,29]; our findings suggest the possibility that work interference with family has more severe impacts in construction projects because experience of such conflict is more salient in the context of temporary projects rather than a permanent organizational environment. The results empirically indicate that Chinese construction professionals might prioritize the work domain over the family domain [5]. This study thus suggests that the two different directions of work–family conflict can result in emotional experiences that deplete resources to varying degrees depending on their different contexts in different industries.

### 5.2. Practical Implications

These findings suggest two important practical implications. First, the mediation analysis suggests that an individual’s emotional resources and emotional experience play important roles in the perception of passive job-related attitudes and engagement in problem behaviors. Thus, the organizations in the construction projects can consider providing some recovery activities at work or after work for project managers and professionals to recoup their emotional resources, such as exercises or lunch breaks [18]. Moreover, these organizations can also provide training programs for owners and contractors to teach them about ways in which emotional regulation can help employees deal with work–family conflict or stress that may result in reduced job satisfaction and increased problem behaviors. For construction professionals, there is a need to acknowledge the internal emotional states, especially negative affect, which may lead to emotional exhaustion. These individuals should learn skills or strategies to alleviate negative affect [56] and manage stress in ways that are suitable to the context of temporary projects.

### 5.3. Limitations and Future Research

Despite these above-mentioned contributions, a number of limitations to the current study should be acknowledged. First, although this study used a time interval to assess independent variables and outcome variables at different time points rather than using a cross-sectional research design, causality should be further explored in these constructs. Future studies should conduct longitudinal research using a long-time interval or an experience sampling method [18,33] in order to robustly capture changes in WFC and affective experiences.

Second, the choice of two mediators (negative affect and emotional exhaustion) was theory-driven (specifically, AET) but there are a number of additional boundary conditions through which WFC may impact individual job-related attitudes and behaviors. Future studies can investigate potential moderators between WFC and job-related attitudes or behaviors. For example, social support may buffer the negative impact of work–family conflict on well-being outcomes [87], and management styles (e.g., servant leadership [88]) can reduce the negative emotional experience induced by work–family conflict to facilitate the positive spillover effect. Moreover, the impact of work–family conflict may varied according to differences among individual traits or personality [30,33], or cultural values [89]. As such, future research could expand the model by incorporating and verifying the potential effects of these important moderators to enrich the work–family conflict research in the project context.

Third, although the self-rating of deviant behavior was recommended because of the fact that employees often performed this behavior in private [90], the leader ratings of individual deviant behavior have been also applied in research on deviance [70]. Hence, future research could employ the employee behavioral ratings from other sources (e.g., supervisor or coworkers) and even apply objective measures (e.g., attendance) to better capture the criterion of deviant behavior.

Finally, this study focused on construction professionals, who were the selected respondents in the sampling procedure, to explore their level of work–family conflict and the emotional response in the project context. Thus, the non-probability sampling method, like the convenience sampling technique, was applied in this study due to the temporal and unique characteristics of construction projects. On the one hand, although the sampling technique was appropriate or convenient to select respondents according to the research purpose for the specific industry [91,92], and this method appears to be common in construction research [93], there may be some issues for convenience sampling, including selection bias or an un-representative sample [94]. Future research could consider using probability-based sampling to reduce the selection bias [95]. On the other hand, the selection of voluntary construction professionals from construction companies or projects using the non-probability sampling technique provides the limitations with regard to the measurement validity and external validity [96]. The measurement validity might be difficult to capture due to the sampling error or bias from the non-probability sampling. The pilot interviews or surveys were recommended to revise and ensure the rationality and quality of the indicators before the formal survey in the future [93]. Further, besides the two-stage survey applied in the study, the mixed method research design is beneficial for overcoming the limitation for the specific populations and facilitate the generalizability to larger samples [93,96]. Specifically, the field experimental designs can be combined to facilitate the external validity and causal inference in the future [97], and the snowball sampling technique can be used to increase the samples to improve the generalizability for larger groups [93].

## 6. Conclusions

Drawing on affective events theory and working from the perspective of emotional resources, the current study demonstrated that there are underlying mechanisms of negative affect and emotional exhaustion in the relationship between work–family conflict and workplace well-being, and between work–family conflict and workplace deviant behavior in the context of construction projects. These findings suggest that there is a significant sequential effect of negative affect and emotional exhaustion mediating the link between work–family conflict and workplace well-being and deviant behavior. This study extends the literature by highlighting the role of emotional experience and by testing affective experience and emotional resource depletion as possible underlying mechanisms for the impact of work–family conflict, and also by comparing the effects of work interference with family and family interference with work in the context of Chinese temporary construction projects.

## Figures and Tables

**Table 1:** The demographic profile of the final valid sample.

Category	*N*	%	Category	*N*	%
Gender			Education		
Male	113	79.0	High school or below	8	5.6
Female	30	21.0	Junior college or undergraduates	129	90.2
Work unit			Postgraduates or above	6	4.2
Real estate company	14	9.8	Occupation		
Construction unit	113	79.0	Construction-site managers	21	14.7
Design institute	2	1.4	On-site operatives	48	33.6
Consulting company	8	5.6	Civil or structural engineers	34	23.8
Others	6	4.2	Cost engineers	30	20.9
			Others	10	7.0
**Information**	**Mean**	**SD**	**Information**	**Mean**	**SD**
Age	30.77	7.35	Organizational tenure	8.03	7.11

Note: SD = standard deviation.

**Table 2:** The results of confirmatory factor analysis.

Models	*χ* ^2^ */df*	CFI	TLI	RMSEA	SRMR
Six-factor model: WIF, FIW, NA, EE, WDB, WWB	1.681	0.916	0.908	0.069	0.060
Five-factor model: WIF + FIW, NA, EE, WDB, WWB	2.491	0.814	0.798	0.102	0.108
Four-factor model: WIF + FIW, NA + EE, WDB, WWB	3.810	0.643	0.619	0.140	0.133
Three-factor model: WIF + FIW, NA + EE + WDB, WWB	4.462	0.558	0.530	0.156	0.145
Single-factor model: WIF + FIW + NA + EE + WDB + WWB	6.348	0.314	0.274	0.193	0.168

Note: WIF = work interference with family, FIW = family interference with work, NA = negative affect, EE = emotional exhaustion, WDB = workplace deviant behavior, WWB = workplace well-being, *χ*^2^ = chi-squared, *df* = degree of freedom, CFI = comparative fit index, TLI = Tucker–Lewis index, RMSEA = root mean square error of approximation, SRMR = standardized root mean square residual.

**Table 3:** The results of descriptive statistics and intercorrelations.

Variables	Mean	SD	1	2	3	4	5	6
1. WIF, T1	3.332	1.020	(0.953)					
2. FIW, T1	2.146	0.872	0.283 **	(0.951)				
3. NA, T1	2.299	0.794	0.470 ***	0.338 ***	(0.921)			
4. EE, T2	2.445	0.926	0.463 ***	0.267 **	0.432 ***	(0.935)		
5. WDB, T2	1.844	0.653	0.189 *	0.300 ***	0.314 ***	0.499 ***	(0.909)	
6. WWB, T2	3.452	0.780	−0.295 ***	−0.112	−0.264 **	−0.356 ***	−0.320 ***	(0.936)

Note: WIF = work interference with family, FIW = family interference with work, NA = negative affect, EE = emotional exhaustion, WDB = workplace deviant behavior, WWB = workplace well-being, SD = standard deviation, T = time. The reliability coefficients are indicated in the diagonals. * *p* < 0.05, ** *p* < 0.01, *** *p* < 0.001.

**Table 4:** The results of regression analysis.

Variables	NA (T1)		EE (T2)		WDB(T2)		WWB(T2)	
	Model 1	Model 1ab	Model 2	Model 2ab	Model 3	Model 3ab	Model 4	Model 4ab
Control variables								
Gender (T1)	0.101	0.098	−0.014	−0.019	0.033	0.040	0.064	0.073
Age (T1)	−0.091	−0.084	−0.019	−0.008	−0.057	−0.077	−0.064	−0.091
Education (T1)	0.090	0.093	0.007	0.014	0.071	0.066	−0.045	−0.051
Attentiveness (T1)	−0.123	−0.127	0.046	0.038	−0.102	−0.096	−0.051	−0.043
EE (T1)			0.147	0.120				
WDB (T1)					0.187 *	0.169	0.161	0.137
WWB (T1)					0.073	0.075	0.538 ***	0.541 ***
Independent variables								
WFC (T1)	0.478 ***		0.286 **		0.222 *		−0.142 *	
WIF (T1)		0.379 ***		0.272**		0.079		−0.213 **
FIW (T1)		0.207 **		0.088		0.222 *		0.073
NA (T1)			0.194 *	0.201*				
*R^2^*	0.300	0.302	0.283	0.288	0.136	0.149	0.298	0.321
*F*	11.747 ***	9.803 ***	7.605 ***	6.765 ***	3.042 **	2.926 **	8.191 ***	7.921 ***

Note: WFC = work–family conflict, WIF = work interference with family, FIW = family interference with work, NA = negative affect, EE = emotional exhaustion, WDB = workplace deviant behavior, WWB = workplace well-being, T = time. The standardized coefficients are presented in the table. * *p* < 0.05, ** *p* < 0.01, *** *p* < 0.001.

**Table 5:** The results of mediation testing (bootstrap = 2000).

Models	Variables	Estimates	Standard Errors	95% BC CI
Model 5	Indirect effects			
	WFC→NA→WDB	0.029	0.041	[−0.058; 0.102]
	WFC→EE→WDB	0.127 **	0.048	[0.054; 0.247]
	WFC→NA→EE→WDB	0.049 *	0.021	[0.019; 0.109]
	WFC→NA→WWB	−0.033	0.068	[−0.177; 0.092]
	WFC→EE→WWB	−0.091 *	0.046	[−0.203; −0.017]
	WFC→NA→EE→WWB	−0.035 *	0.018	[−0.089; −0.009]
Model 6	Indirect effects			
	WIF→NA→WDB	0.022	0.023	[−0.030; 0.064]
	FIW→NA→WDB	0.014	0.017	[−0.013; 0.057]
	WIF→EE→WDB	0.094 **	0.033	[0.043; 0.182]
	FIW→EE→WDB	0.032	0.035	[−0.030; 0.107]
	WIF→NA→EE→WDB	0.030 *	0.014	[0.011; 0.071]
	FIW→NA→EE→WDB	0.019 *	0.009	[0.006; 0.052]
	WIF→NA→WWB	−0.016	0.040	[−0.011; 0.052]
	FIW→NA→WWB	−0.010	0.026	[−0.068; 0.040]
	WIF→EE→WWB	−0.061 *	0.030	[−0.146; −0.005]
	FIW→EE→WWB	−0.020	0.024	[−0.085; 0.015]
	WIF→NA→EE→WWB	−0.019 *	0.009	[−0.053; −0.004]
	FIW→NA→EE→WWB	−0.012 *	0.006	[−0.042; −0.002]

Notes: WFC = work–family conflict, WIF = work interference with family, FIW = family interference with work, NA = negative affect, EE = emotional exhaustion, WDB = workplace deviant behavior, WWB = workplace well-being, BC CI = bias-corrected confidence interval. * *p* < 0.05, ** *p* < 0.01.

**Table 6:** The results of hypothesis testing.

Hypothesis	Coefficients	Results
Hypothesis 1. WFC→WDB	0.222 * (Model 3)	Support
• Hypothesis 1a. WIF→WDB	0.079 (Model 3ab)	Unsupport
• Hypothesis 1b. FIW→WDB	0.222 * (Model 3ab)	Support
Hypothesis 2. WFC→WWB	−0.142 * (Model 4)	Support
• Hypothesis 2a. WIF→WWB	−0.213 ** (Model 4ab)	Support
• Hypothesis 2b. FIW→WWB	0.073 (Model 4ab)	Unsupport
Hypothesis 3. NA→EE	0.194 * (Model 2)	Support
Hypothesis 4. WFC→NA→WDB	0.029 (Model 5)	Unsupport
• Hypothesis 4a. WIF→NA→WDB	0.022 (Model 6)	Unsupport
• Hypothesis 4b. FIW→NA→WDB	0.014 (Model 6)	Unsupport
Hypothesis 5. WFC→NA→WWB	−0.033 (Model 5)	Unsupport
• Hypothesis 5a. WIF→NA→WWB	−0.016 (Model 6)	Unsupport
• Hypothesis 5b. FIW→NA→WWB	−0.010 (Model 6)	Unsupport
Hypothesis 6. WFC→EE→WDB	0.127 ** (Model 5)	Support
• Hypothesis 6a. WIF→EE→WDB	0.094 ** (Model 6)	Support
• Hypothesis 6b. FIW→EE→WDB	0.032 (Model 6)	Unsupport
Hypothesis 7. WFC→EE→WWB	−0.091 * (Model 5)	Support
• Hypothesis 7a. WIF→EE→WWB	−0.061 * (Model 6)	Support
• Hypothesis 7b. FIW→EE→WWB	−0.020 (Model 6)	Unsupport
Hypothesis 8. WFC→NA →EE→WDB	0.049 * (Model 5)	Support
• Hypothesis 8a. WIF→NA→EE→WDB	0.030 * (Model 6)	Support
• Hypothesis 8b. FIW→NA→EE→WDB	0.019 * (Model 6)	Support
Hypothesis 9. WFC→NA →EE→WWB	−0.035 * (Model 5)	Support
• Hypothesis 9a. WIF→NA→EE→WWB	−0.019 * (Model 6)	Support
• Hypothesis 9b. FIW→NA→EE→WWB	−0.012 * (Model 6)	Support

Notes: WFC = work–family conflict, WIF = work interference with family, FIW = family interference with work, NA = negative affect, EE = emotional exhaustion, WDB = workplace deviant behavior, WWB = workplace well-being, * *p* < 0.05, ** *p* < 0.01.
